# Mechanical Behavior and Healing Efficiency of Microcapsule-Based Cemented Coral Sand under Various Water Environments

**DOI:** 10.3390/ma14195571

**Published:** 2021-09-25

**Authors:** Yue Qin, Qiankun Wang, Dongsheng Xu, Wei Chen

**Affiliations:** School of Civil Engineering and Architecture, Wuhan University of Technology, Wuhan 430070, China; yqin@whut.edu.cn (Y.Q.); wangqk@whut.edu.cn (Q.W.); chen_wei@whut.edu.cn (W.C.)

**Keywords:** CCS, microcapsule, mechanical properties, self-healing, water environment

## Abstract

The cracks in the cemented coral sand (CCS) would result in significant damage for the marine structures. In this study, the effective and efficiency of microcapsules in self-healing CCS under various water environments were investigated with a series of experimental tests. Firstly, a new preparation method was proposed to fabricate the microcapsules with a wide particle size distribution, which was adapted to the high porosity, large difference in pore size, and uneven distribution of CCS. Secondly, the mechanical properties of microcapsule-based CCS were examined by the uniaxial compressive tests and split Hopkinson pressure bar (SHPB) tests. The results indicated that the microcapsule could improve the initial strength of CCS. The CCS mixed with 3% of the microcapsule that synthesized under a rotating speed of 450 rmp had the highest compressive strength at the initial strain state. Finally, the healing efficiency of microcapsule for CCS was investigated in various environmental conditions, which were freshwater, seawater, and water of various pH values. The non-destructive experiment approach of the piezoelectric transducer (PZT) test was adopted to evaluate the healing efficiency of microcapsules. Experimental results indicated that the healing efficiency of microcapsules in freshwater and seawater were 75% and 59.56%, respectively. In contrast, the acid and alkali water environment would greatly reduce the healing efficiency of microcapsules in CCS.

## 1. Introduction

The coral sand was widely used locally as a construction material at the lower latitudes. The concrete with coral sand is different from that using silicon sand, as the coral sand has many inherent characteristics, such as high internal void ratio, particle breakage, and irregular particle shape [1,2,3]. The major components of coral sand and silicon sand are CaCO_3_ and SiO_2_, respectively. Thus, the coral sand has lower compressive strength than the silicon sand [4,5]. Thus, the concrete based on coral sand should inevitably focus on the internal cracks due to the particle breakage of coral sands [6,7].

In recent decades, many approaches have been proposed for the healing of cracks. The healing method can be classified as two categories, which are the passive reinforcement and active healing approaches. The passive reinforcement method was referred to add polyvinyl alcohol fiber (PVA) or steel slag fiber into the concrete mortar to improve the strength of the concrete, which would restrain the crack development in the concrete [8]. The active healing method, also called self-healing technology, has attracted wide attentions in recent years, and many approaches have been proposed, such as the osmotic crystallization method [9], microcapsule method [10,11,12,13,14], liquid-core fiber/fiber method [15], microbial method [16,17], and shape memory alloy method [18,19,20,21,22]. Among these methods, the microcapsule method is widely adopted due to the effectiveness and efficiencies.

White et al. first reported microencapsulation as an ideal carrier to encapsulate and protect the remediation agent from the influence of the external environment and processing process [23]. Since then, microcapsule self-healing has attracted global attention. The encapsulated dicyclopentadiene (DCPD) in a urea–formaldehyde shell was used with ruthenium-based Grubbs catalyst for the healing of epoxy resin matrix. Rule et al. studied the influence of the particle size of microcapsules on the healing effect of composite materials [24]. The healing object was epoxy resin conical double cantilever beam (TDCB), which was revealed under certain conditions. Tan et al. carried out the characterization of the healing efficiency of self-healing polymers based on the continuum damage model, and they introduced the index of energy as the reference amount of the healing efficiency [25]. Li et al. explored the synthesis process parameters of microcapsules and analyzed the physical properties of microcapsules [26]. Ou et al. studied the critical failure stress of microcapsules [27]. Yuan et al. pointed out that the microcapsule can bear the external force in the process of composite with the matrix material, and it has a good bond with the matrix material [28]. However, fewer studies focus on the effectiveness of microcapsules in various sea water conditions [29,30].

The split Hopkinson pressure bar (SHPB) test is an effective experimental method to study the dynamic mechanical properties of concrete or the similar materials under a one-dimensional stress state, which can directly test the relationship between the stress and strain of materials at high strain rates (100–5000 s^−1^) [31,32,33]. The split Hopkinson pressure bar was designed by Kolsky in 1949 [34]. The SHPB tests are based on assumption of the one-dimensional elastic wave and the quasi-static equilibrium. Many dynamic mechanical parameters such as dynamic compression [35], dynamic tensile [36], dynamic shear [37], and crack growth rate [38] could be determined by modifying the devices.

The internal crack identification is difficult due to the experimental limitations. Some non-destructive approaches such as the piezoelectric transducer (PZT) [39,40,41], nuclear magnetic resonance [42,43], acoustic emission [44,45], and industrial computerized tomography CT [46,47,48,49] were adopted to quantify the internal structure or the crack of the material. The PZT test is the most commonly used method for its advantages of being economical, fast, and easy to operate.

Coral sand has characteristics of irregular particle shape, uneven particle size, and large aspect ratio, which result in large skeleton pores and an uneven distribution of pore structure. Based on the void properties of coral sand mortar, this study proposed the controlled particle size distributions of the microcapsule by considering the filling effect of a microcapsule. Therefore, a series of cemented coral sand (CCS) mortar specimens with urea–formaldehyde resin microcapsules were prepared. The mechanical behavior of CCS specimens was examined with the uniaxial compressive tests and split Hopkinson pressure bar (SHPB) tests. In addition, the non-destructive testing approach by using the piezoelectric transducer (PZT) was adopted to examine the healing efficiency of a microcapsule on the CCS in freshwater, seawater, and various acid and alkali water environments.

## 2. Materials and Test Procedures

### 2.1. Material

**Figure 1:**
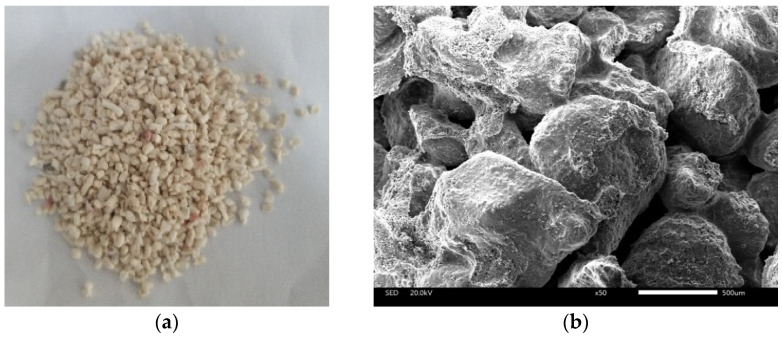
Particle morphology of coral sand: (**a**) picture of coral sand; (**b**) SEM image with 50 × magnification.

The coral sand used in this study was obtained from the South China Sea. The major components of the coral sand were aragonite and magnesium calcite. The equivalent calcium carbonate content was more than 90%. Figure 1 shows the particle morphology of coral sand and SEM results. Figure 1b shows that the biogenic coral sand particles have the characteristics of high porosity and sharp edges, leading to the large difference in pore size and uneven distribution of the sand.

Table 1 lists the particle size of coral sand used in the following tests. Figure 1 shows the particle morphology and SEM results of coral sand. In this study, the particle size of coral sand was ranged from 0.25 to 1 mm, which was classified as fine sand. The mass percentage of particle from 0.25 to 0.5 mm was 43.62%, and that from 0.5 to 1 mm was 56.38%. 

The cement used in this study was ordinary Portland cement (P.P. 42.5) provided by Huaxin Cement Co., Ltd. in China. Freshwater and artificial seawater were prepared according to the ASTM standard [50]. The dosage of each component per liter of artificial seawater is shown in Table 2.

### 2.2. Microcapsules

The microcapsules fabricated in this study were epoxy resin coated with urea–formaldehyde resin. The materials required for the synthesis of microcapsules mainly included core materials and capsule wall materials. The raw materials were epoxy resin (E-51), urea, formaldehyde, triethanolamine, anhydrous sodium carbonate, sodium dodecyl benzene sulfonate, polyvinyl alcohol (PVA), octanol, ammonium chloride, and resorcinol. Table 3 lists the detailed information. The synthesis method was in situ polymerization including PH adjustment stage, emulsifying stage, defoaming stage, acidizing stage, and wall curing stage; the detailed synthesis process is as follows. First, 15 g of urea was added into 40.5 g of formaldehyde solution, which was followed by adjusting the pH within 8 to 9 with triethanolamine. The mixture solution was heated to 70 °C and stirred for an hour to get the prepolymer. The epoxy resin, sodium dodecyl benzene sulfonate, and polyvinyl alcohol (PVA) were mixed with freshwater. Then, the obtained solution was dispersed for 30 min at a constant temperature of 70 °C. The n-octanol was added to remove the foam produced during the emulsification. The prepared prepolymer and emulsion were mixed at a specific rotating speed to obtain microcapsules with various particle grades. After that, NH_4_Cl (catalyst) was slowly added to the solution and stirred for 2 h to keep the pH within 2 to 3 (acidification treatment). The resorcinol was added to the mixture solution followed by curing for 3 h at a bath temperature of 70 °C. Subsequently, the 2% NaCO_3_ solution was added to the mixture, which was controlled with the pH of 7.0. The final microcapsules were obtained after drying at a constant temperature of 25 °C.

**Figure 2:**
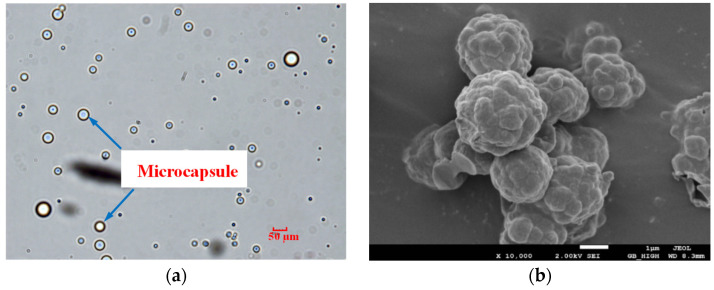
Images of microcapsule: (**a**) optical microscope image; (**b**) SEM image.

The prepared microcapsules were evenly dispersed without agglomeration observed under an optical microscope. Spherical microcapsules were observed through SEM images, the capsule shell with a rough surface was dense and free of voids. The optical microscope image and SEM image are shown in Figure 2. The particle size distribution of microcapsules was analyzed by the Laser Particle Size Analyzer (Betters E 2000) with a scan range of 0.1–2000 μm. The size distribution of the microcapsules was 0–800 μm, and the density distribution of particle size was normally distributed. This method produced specific grades of microcapsules, which broke through the limitation of the traditional healing method of using a single particle size microcapsule. The prepared microcapsules in this study contributed to the excellent structure and performance of CCS.

### 2.3. Specimen Preparation

**Figure 3:**
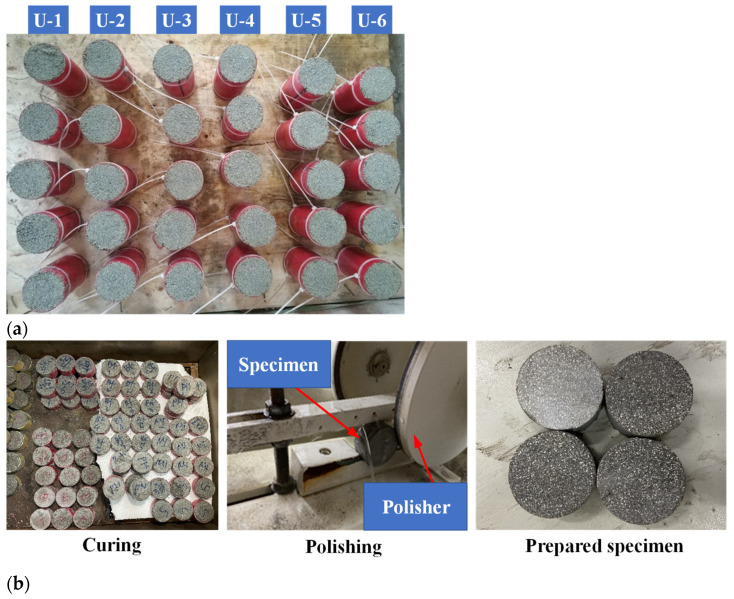
Prepared CCS specimens: (**a**) typical specimens for UCS tests; (**b**) specimens for the SHPB tests.

Considering the significant water absorption capacity of coral sand due to its abundant internal pores, the water–cement ratio of the CCS was 1:1. The cement and water contents were both 9.68%. The mass proportion of coral sand was 80.64%. Microcapsules and catalysts were mixed together when preparing the microcapsule-based CCS. The catalyst was DMP-30, whose dosage was half of the microcapsules in mass. The mixture was shaped into cylinder specimens by PVC pipes. The CCS specimen with a dimension of 50 mm in diameter and 100 mm in height were prepared for the uniaxial compressive tests. For the SHPB tests, the dimension was 50 mm in diameter and 25 mm in height. Specially, the CCS specimen with dimensions of 50 mm in diameter and 100 mm in height were reserving a small groove (i.e., with dimensions of 10 mm × 3 mm × 8 mm) at each end for the installation of two pieces of PZTs. The specimens were cured for 1 day under natural conditions and then placed in the cement rapid curing box at 75 °C for 3 days. Finally, the specimens were demolded after cooling and were placed under natural conditions for 1 day. For the seawater mixed specimen, the preparation and maintenance steps were the same except replacing the above freshwater with seawater. Table 4 shows the detailed preparation of CCS specimens under different experimental tests. Figure 3 shows the typical prepared CCS specimens. In this study, the specimens used for the uniaxial compressive test were labeled as U-1 to U-8 with various microcapsule contents. Specimens labeled as S-1 to S-3 and P-1 to P-7 were used for the SHPB and PZT tests, respectively. A total of 18 groups of specimens were prepared to investigate the healing efficiency under various water environments. 

### 2.4. Test Procedures

#### 2.4.1. Uniaxial Compressive Test

Uniaxial compressive tests were carried out for CCS to evaluate the effect that microcapsules had on the initial compressive performance. In this study, displacement control mode was adopted with a loading speed of 0.08 mm/min. The failure load and the corresponding displacement were recorded to obtain the stress–strain curve and the failure picture of mortar, through which the strength of the mortar under different conditions was determined.

#### 2.4.2. SHPB Test

**Figure 4:**
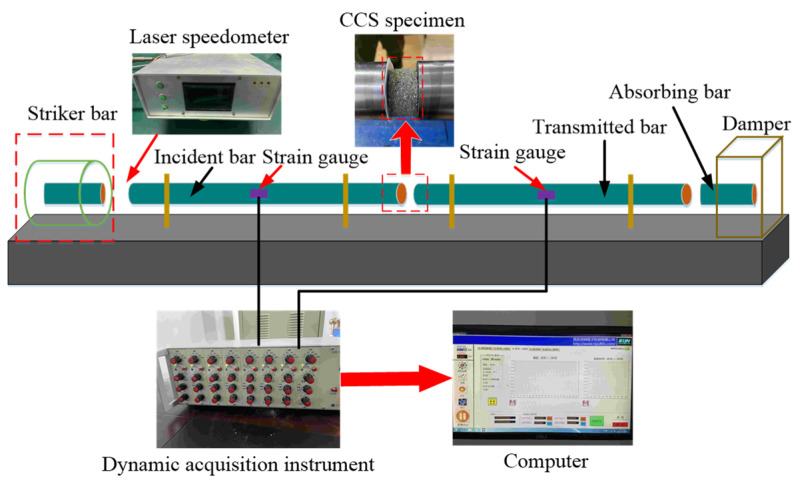
Diagram of the SHPB test system.

The schematic diagram of the SHPB system is shown in Figure 4. The required test pressure was provided by the pressure pump with the air compressor of the launching system, which was controlled by a computer. The impact velocity of the bullet was recorded by the laser speedometer. The reading of the hyper dynamic acquisition instrument was set to zero before the SHPB test. The air pressure of the exhaust valve was set through the computer, and the impact bar was released when the pressure stabilized to the preset value. The speed of the impact bar, strain of the incident bar, and transmission bar were automatically collected by the computer during impaction. The failure pattern of the specimens was photographed by a camera for subsequent analysis. The parameters of the SHPB are shown in Table 5.

In this study, the SHPB test results were analyzed by the three-wave method. In this approach, the stress, strain, and strain rate of the CCS specimens could be deduced according to one-dimensional stress assumptions, stress wave propagation theoretical assumptions, and the continuity requirement of displacement. The expressions are as follows:

(1) $$\sigma_{s}=\frac{\left(\varepsilon_{i}{+\varepsilon }_{r}{+\varepsilon }_{t}\right)E_{0}A}{{2A}_{s}}$$

(2) $${\dot{\varepsilon }}_{s}=\frac{\left(\varepsilon_{i}{-\varepsilon }_{r}{-\varepsilon }_{t}\right)C_{0}}{L_{s}}$$

(3) $$\varepsilon_{s}=\int_{0}^{t}{\dot{\varepsilon }}_{s}dt$$

where *E*_0_, *C*_0_, *A*, and *A_S_* are the elastic modulus, wave velocity, cross-sectional area of the bar, and the cross-sectional area of the specimen, respectively; *ε_i_*, *ε_r_*, and *ε_t_* are the strain of the incident bar, reflection bar, and transmission bar during the impact process, respectively; *L_S_* is the length of the specimen. According to the stress balance condition, the specimens were considered to reach the stress balance when the unbalanced stress was less than 5% [8], which can be expressed as:

(4) $$R=\frac{\sigma_{i}{+\sigma }_{r}{-\sigma }_{t}}{\left(\sigma_{i}{+\sigma }_{r}{+\sigma }_{t}\right)/2}\leq 5\%$$

where *σ_i_*, *σ_r_*, and *σ_t_* are the incident stress, reflected stress, and transmitted stress, which can be obtained through the SHPB tests. The incident energy, reflected energy, and transmitted energy of the specimen can be expressed as follows:

(5a) $$W_{i}=\frac{AC_{0}}{E_{0}}\int_{0}^{t}\sigma_{i}^{2}dt$$

(5b) $$W_{r}=\frac{{\mathrm{AC}}_{0}}{E_{0}}\int_{0}^{t}\sigma_{r}^{2}dt$$

(5c) $$W_{t}=\frac{{\mathrm{AC}}_{0}}{E_{0}}\int_{0}^{t}\sigma_{t}^{2}dt$$

where *W_i_*, *W_r_*, and *W_t_* are the incident energy, reflected energy, and transmitted energy, respectively; *t* is the duration of the stress wave. The dissipated energy or absorbed energy of the specimen can be expressed as *W_ed_* = *W_i_* − *W_r_* − *W_t_*. The energy density can be approximately expressed as:

(6) $$w_{d}=\frac{W_{ed}}{V}$$

where *w_d_* is the energy density, and *V* is the volume of the specimen.

### 2.5. PZT Test

The PZT test was conducted to compare the specimens of before and after healing to evaluate the healing effect. The arbitrary waveform generator (SDG1012X) was adopted as the PZT signal generating equipment. The generating signal was a single cycle sine wave with the voltage and frequency of 20 Vpp and 7 kHz, respectively. The charge amplifier (YE5852B) was adopted as the signal amplifier with the amplification of 1000 times. The oscilloscope was the model of SDS1104-X. The transmitted and received signals in the PZT tests were time-domain signals, which were transformed into frequency domain signals through Fast Fourier Transform (FFT) method. Then, the energy of the signals was calculated by integrating in the frequency domain according to:

(7) $$E=\int_{{}_{t_{1}}}^{{}_{t_{2}}}u^{2}dt$$

where *t*_1_, *t*_2_ are the start time and end time of the signal; *u* is the amplitude of the signal. In this study, the incident energy of all the specimens was the same because all the incident signals were of the same frequency and amplitude. In order to evaluate the healing effect of microcapsules on CCS, the specimens were pre-damaged. The core material of microcapsules was released during the compressive process. The specimens were repaired and strengthened when the core material penetrated and cemented in the cracks and pores. For each specimen, the PZT tests were performed three times, which are before compression, after pre-damage, and after healing. The energy of the received signals of specimens at each time point was calculated according to Equation (7). The energy change could be an indicator to reflect the internal crack and the healing information of the CCS specimens. The healing efficiency can be expressed as:

(8) $$\eta =\frac{E_{2}-E_{1}}{E_{0}-E_{1}}\times 100\%$$

where *E*_0_, *E*_1_, and *E*_2_ are the signal energy of the specimens at the state of undamaged, damaged, and repaired, respectively. The difference of specimens repaired in various water environments were studied. After treatment of pre-damage, the specimens were soaked in different water environments and placed in a standard curing box for 2 days. Then, the healing effect was evaluated by conducting the PZT tests on specimens after drying. The performance of microcapsules on CCS repaired in seawater, freshwater, and water of different pH values was compared. The CCS specimens were completely immersed in solution during the curing process. A type of covered container with small holes was designed to avoid the change of solution concentration caused by water evaporation during the curing process and avoid bloating caused by excess steam.

## 3. Mechanical Properties of Microcapsule-Based CCS

### 3.1. Uniaxial Compressive Strength (UCS)

**Figure 5:**
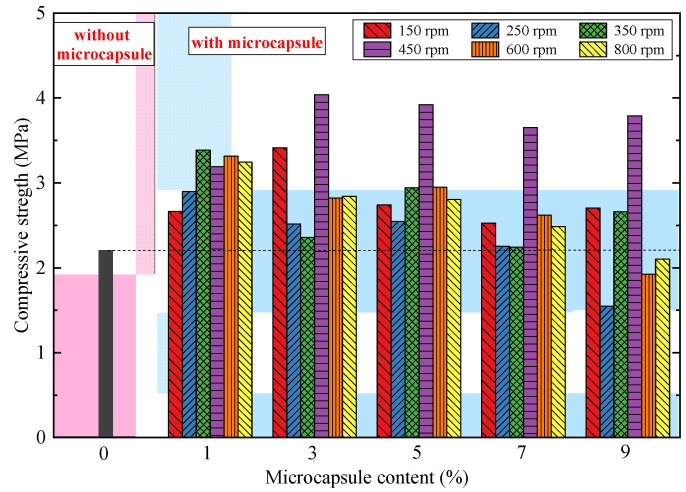
Compressive strength versus microcapsule content.

Microcapsules could affect the initial mechanical properties of CCS apart from the self-healing effect. Thus, the effects of mixed content and particle size distribution of microcapsules on the mechanical properties of CCS were studied. Five groups of CCS specimens with mass ratios of microcapsules to cement of 1%, 3%, 5%, 7%, and 9% were prepared and tested. In order to investigate the effect of particle size of microcapsules, six grain groups of microcapsules were fabricated under various rotating speeds, such as 150 rpm, 250 rpm, 350 rpm, 450 rpm, 600 rpm, and 800 rpm. Uniaxial compressive tests were conducted on CCS with different contents and grain groups of microcapsules. The compressive strength results of the six groups of microcapsule-based CCS were compared with that of the specimen without a microcapsule, as shown in Figure 5. The initial strength of specimens was improved effectively with microcapsules when the content was less than 9%. Especially, the microcapsules prepared under a rotating speed of 450 rpm had the most significant effect on the strength of the specimen with the strength growth rate ranging from 45% to 83%. The strength reached the maximum when the microcapsule content was 3%. Coral sand has an abundance of internal pores. An appropriate incorporation of microcapsules could fill the pores of the specimen skeleton, increasing the compactness and compressive strength [28]. Therefore, the optimization function of microcapsules was better on the initial strength of CCS than that of other cemented material.

As can be seen from Figure 5, the strength of the specimen with a microcapsule content of 9% was lower than that of specimens without microcapsules. It was because only a part of the microcapsules filled the pores, while the other part occupied the space of the cemented matrix or coral sand particles with the excessive number of microcapsules. For the microcapsules that occupied the space of the coral sand particle condition, the microcapsules built a new skeleton with the sand particles. The surface morphology and material properties of microcapsules determine that the bonding strength between microcapsules and sand particles was significantly lower than that between sand particles, which weakened the strength of the specimens. The Young’s modulus of microcapsules was much smaller than that of the cemented matrix. Thus, the microcapsules occupied the space of the cemented matrix, which was equivalent to add artificial pores inside the specimen. Therefore, it would result in the reduction of compressive strength of the CCS specimens.

### 3.2. Comparisons of Seawater and Freshwater

**Figure 6:**
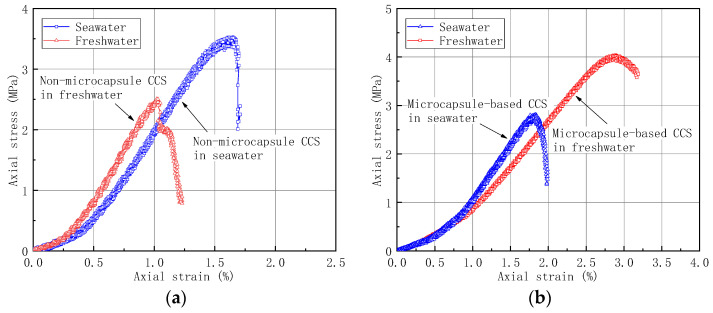
Comparison of compressive strength of freshwater mixed and seawater mixed specimens: (**a**) microcapsule content = 0%; (**b**) microcapsule content = 3%.

The maximum initial strength was obtained when the microcapsule synthesis rate was 450 rpm and the microcapsule content was 3%. It indicates that the CCS was in the ideal state of compactness and intergranular bite strength under the condition. Considering that the coral reef engineering structure would be subject to various salinities of seawater, the mechanical properties of microcapsule-based CCS specimens were investigated in seawater conditions to analyze the effect of salinity on the mechanical properties. Figure 6 shows the compressive curves of microcapsule-based CCS specimens prepared in seawater and freshwater conditions. The microcapsule content in Figure 6a and Figure 6b were 0% and 3%, respectively. 

Under the freshwater condition, the strength increased with the addition of microcapsules. However, in seawater condition, it has the opposite phenomenon, which is that the compressive strength of CCS specimens decreased with the increase of microcapsules. The reason may be attributed to the effect of cation in seawater on the viscosity of urea–formaldehyde resin. The wall material of the microcapsule would further affect the strength of the CCS. In this study, the wall material of the microcapsule is urea–formaldehyde resin whose molar ratio of urea and formaldehyde was 1:2. The gelation of urea–formaldehyde resin was a process of colloidal particle coalescence and aggregation structure development. For the seawater mixed specimens, the charge of Na^+^ and Mg^2+^ ions were opposite to those of the urea–formaldehyde resin granules. According to the colloidal stability theory (DLVO theory), part of the Na^+^ and Mg^2+^ cations in seawater entered the adsorption layer of colloidal particles, resulting in the decrease in the absolute value of zeta potential and eventually tending to zero. The system viscosity increased as the repulsive barrier between particles decreased. Thereby, the strength of the seawater mixed specimens decreased by 44% compared with the freshwater mixed ones.

### 3.3. SHPB Test Results

**Figure 7:**
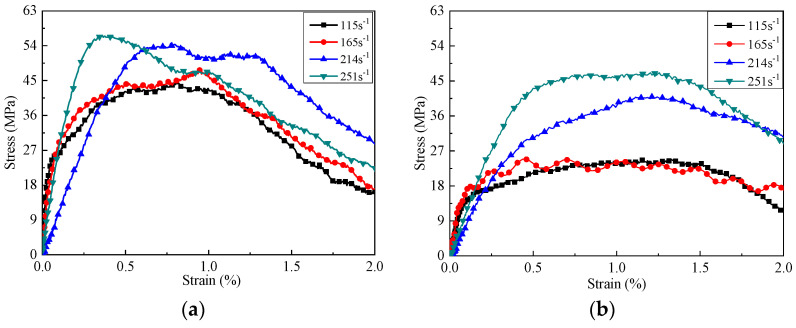
Stress–strain curves: (**a**) microcapsule-based specimen; (**b**) non-microcapsule specimen.

**Figure 8:**
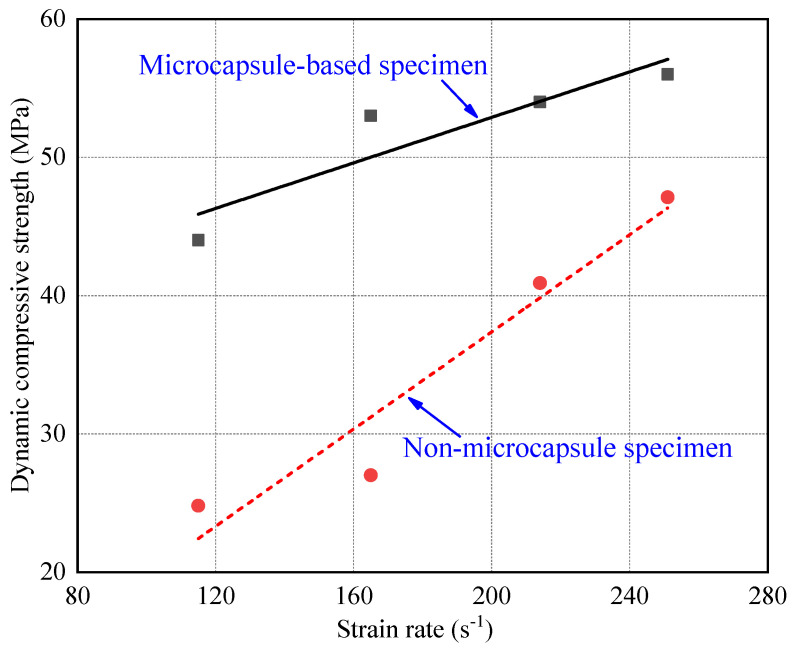
Relation between dynamic compressive strength and strain rate.

The strain rate of the specimens under impact pressure of 0.6 Bar, 0.8 Bar, 1.0 Bar, and 1.2 Bar were 115 s^−1^, 165 s^−1^, 214 s^−1^, and 251 s^−1^, respectively. It can be seen from Figure 7 that the failure mode of CCS under impact load belonged to brittle failure. The evolution of the deformation failure of CCS was the process of growth and the coalescence of internal cracks. However, under the condition of high strain rate, there was not enough time for the extension and coalescence of cracks. Thereby, the stress-bearing capacity increases with the strain rate. In Figure 7, the experimental dynamic peak stress changed slightly when the strain rate was lower than 200 s^−1^ and increased obviously when the strain rate grew higher than 200 s^−1^. According to Pajak et al. [51], it was associated with the inertial effects under high strain rate. In addition, it quickly reached the peak stress for the microcapsule-based specimens when the strain was less than 1%. Microcapsules increased the dynamic compressive strength of CCS. The reason was that the microcapsules blocked the path of crack penetration, which shortened the evolution of deformation. The decrease of deformation further increased the dynamic compressive strength. Figure 8 shows the peak strain under various stain rates. It can be seen that the peak strain of the microcapsules-based CCS was less than that of specimens without microcapsules.

**Figure 9:**
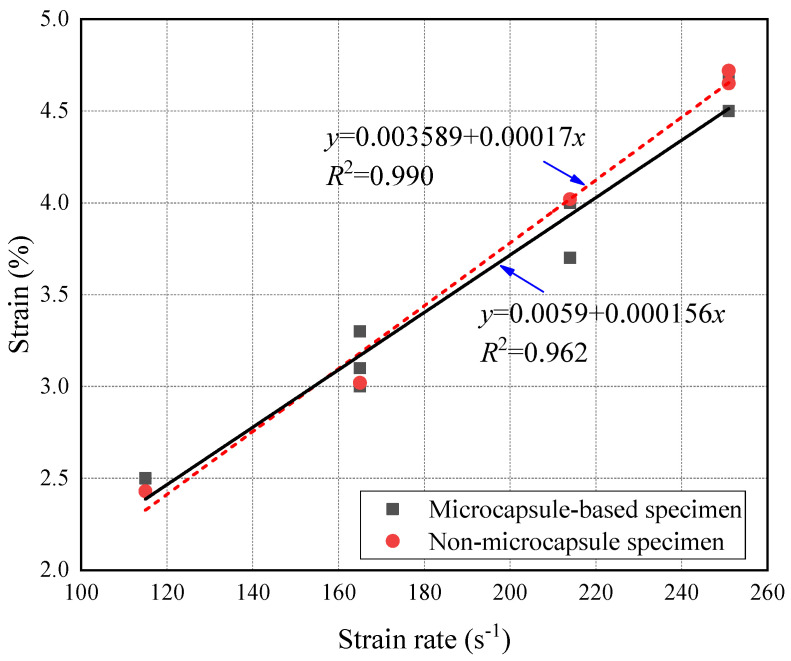
Peak strain under various strain rates.

**Figure 10:**
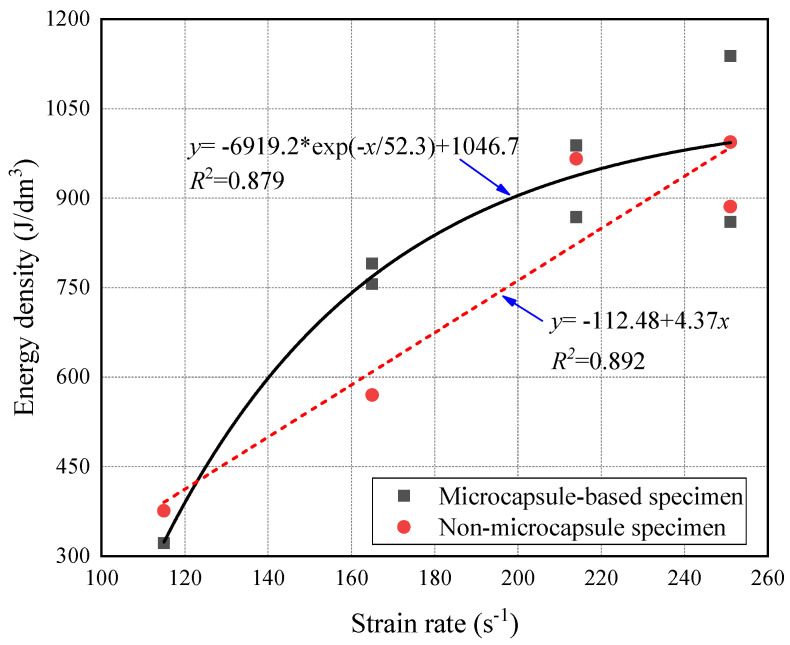
Energy density under various strain rates.

**Figure 11:**
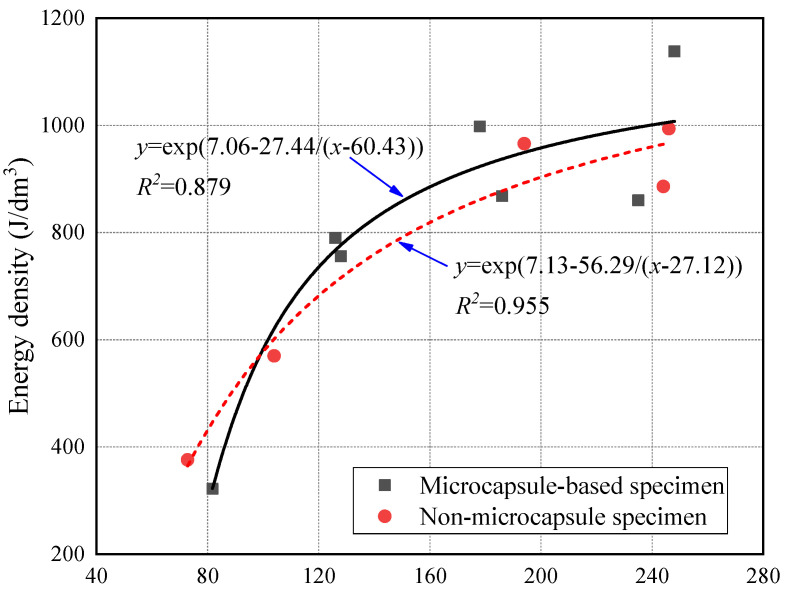
Energy density varied with incident energy.

Figure 9 shows the relationship between energy density and strain rate of CCS. Both the value and dispersion of energy density for microcapsule-based CCS was slightly higher than that for specimens without microcapsules under the high strain rate. It was because the random distribution of microcapsules in CCS increased the difference of the internal structure. Figure 10 shows the relationship between energy density and incident energy. It can be found that the energy density has an exponential relationship with the incident energy. The energy density increased with incident energy, and the growth rate was gradually slowed down. It indicates that the energy absorption ability of CCS was closely related to the internal structure and the energy was absorbed by both the skeleton and cement matrix of the mortar specimen. The incident energy was absorbed preferentially by the more vulnerable mortar skeleton. With the increase of incident energy, the main body of energy absorption changed from a single energy absorption pattern to a joint energy absorption pattern. The growth rate of the energy density slowed down as the change of the main body of energy absorption. The energy density tended to be stable when the incident energy exceeded the energy absorption threshold of the skeleton and cement matrix. Subsequently, the further increase in incident energy mainly contributed to the damage degree of the specimen. As can be seen from Figure 11, the energy density of microcapsule-based specimens was higher than that of specimens without microcapsules, while the growth rate was lower when the incident energy was less than 130 J. Meanwhile, the energy density of the two types of specimens increased at almost the same rate when the incident energy was higher than 130 J. The above results show that the incorporation of microcapsules mainly affected the energy absorption efficiency of the skeleton while having little effect on the cement matrix.

**Figure 12:**
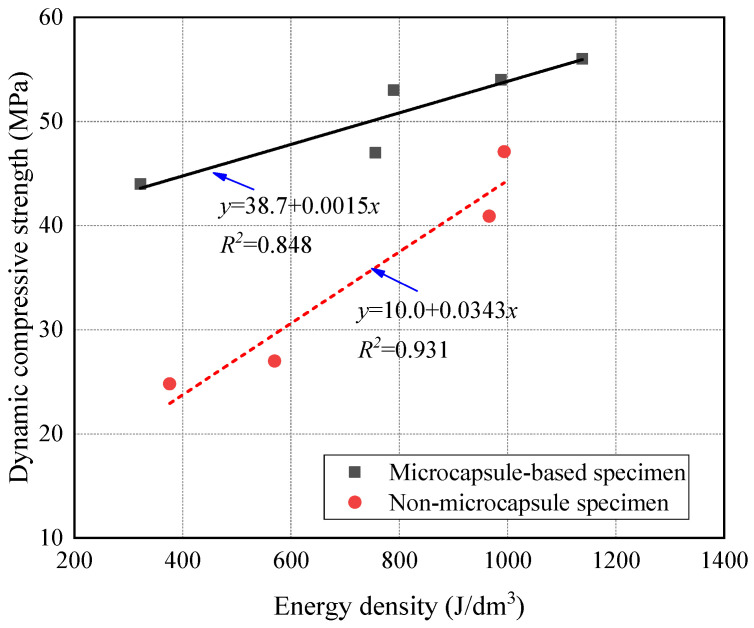
Relationship between dynamic compressive strength and energy density.

Figure 12 shows that there was a positive linear correlation between dynamic compressive strength and energy density. The absorbed energy was mainly dissipated in the damage and deformation of the skeleton and cement matrix. Thereby, the deformation was closely related to the change of energy. The deformation hysteresis appeared with the increase of absorbed energy, which improved the dynamic compressive strength of the specimens. Therefore, the energy density could reflect the essential characteristics of dynamic compressive strength of the CCS. As observed from Figure 12, the dynamic compressive strength improved with the incorporation of microcapsules and energy density. The growth rate of the microcapsule-based specimens was half of that without microcapsules. It indicates that part of the energy absorbed by the microcapsules was dissipated in the damage and deformation, and the other part was consumed in the heat change of the microcapsule itself. The microcapsules reduced the dependence of the dynamic compressive strength on the incident energy. The growth rate that the dynamic compressive strength varied with energy density was lower for the microcapsule-based CCS.

Table 6 shows the comparison on the failure modes under different strain rates between the microcapsule-based specimens and the specimens without microcapsules. It can be seen from Table 1 that the damage degree of specimens increased with the strain rate. The CCS exhibited obvious splitting failure characteristics at the low strain rate. The fragments of damaged specimens changed gradually from massive to granular and at last powder as the strain rate increased. The damage degree of the microcapsule-based specimens was less than that of the specimens without microcapsules. Especially at the low strain rate, the fragments of the microcapsule-based specimens were more complete compared with the one without microcapsules. The difference was small at the high strain rate.

## 4. Healing Efficiency of Microcapsule in CCS 

### 4.1. Healing Efficiency in Freshwater and Seawater Environment

The healing efficiency of microcapsule-based CCS healing in freshwater and seawater was compared by the PZT tests. Each specimen underwent three PZT tests that were conducted at the stages of undamaged before compression, pre-damage of 1 mm deformation, and post-healing, respectively. The received signal before and after the burst of microcapsules was analyzed according to Equation (7). The healing efficiency of microcapsules was evaluated by Equation (8). According to the uniaxial compression test results, it was found that there was a 0.62 mm residual deformation for the specimen after being compressed to 1 mm and unloaded. It indicated that the plastic deformation of specimens occurred due to internal damage under the compression deformation of 1 mm; the microcapsules were thought to burst under that condition. Therefore, the compression deformation of 1 mm was selected as the standard of pre-damage of the CCS specimen in the PZT test. 

**Figure 13:**
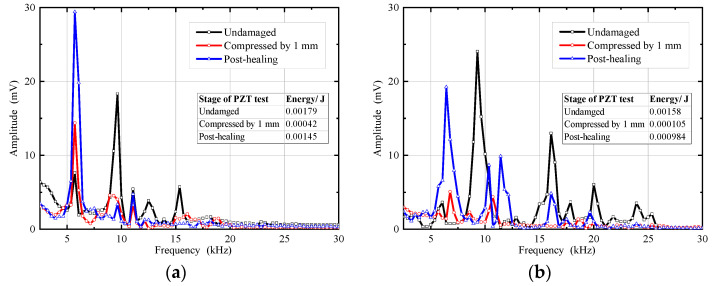
Frequency spectrum for microcapsule-based CCS specimens: (**a**) in freshwater; (**b**) in seawater.

Figure 13 shows the frequency spectrum of specimens before and after restoration by microcapsules in the working environment of freshwater and seawater. The healing efficiency was 75.18% and 59.56% in freshwater and seawater, respectively. The results indicate that the microcapsules have an obvious healing effect on the CCS and have good application prospects in self-healing of damaged structures for coral reef engineering. The healing efficiency in freshwater was better than in seawater, which was associated with the viscosity change of the wall material of the microcapsules in seawater.

### 4.2. Healing Efficiency in Solution of Various pH

**Figure 14:**
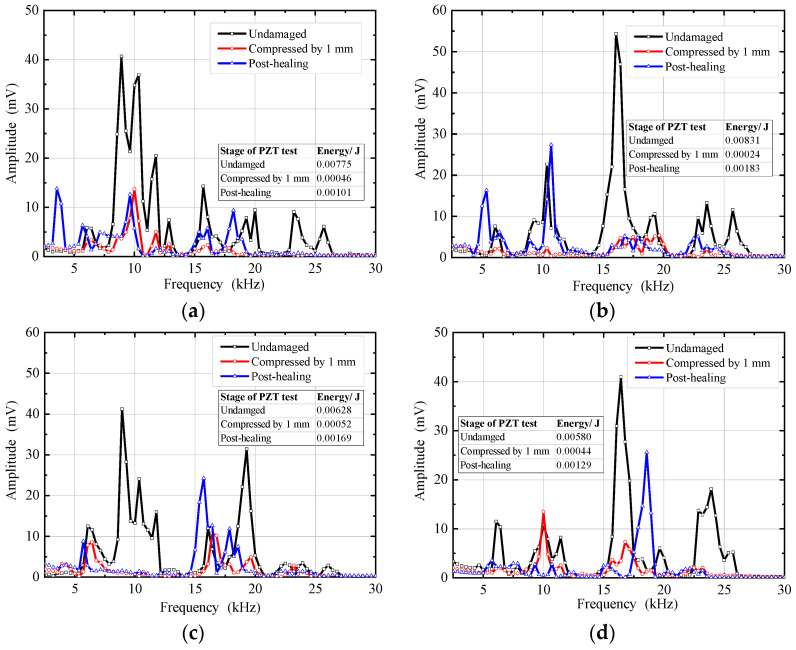

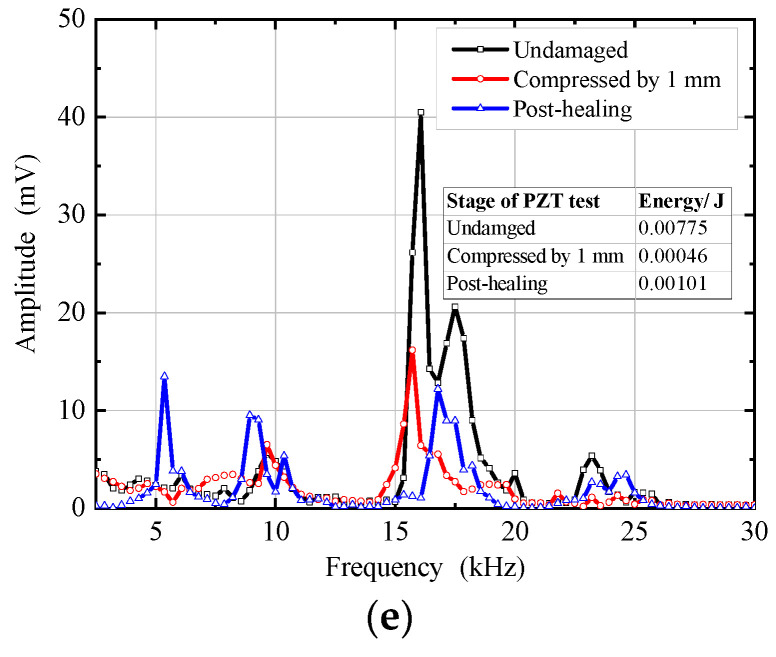
Frequency spectrum of microcapsules in various water environments: (**a**) pH = 1; (**b**) pH = 3; (**c**) pH = 5; (**d**) pH = 9; (**e**) pH = 11.

**Figure 15:**
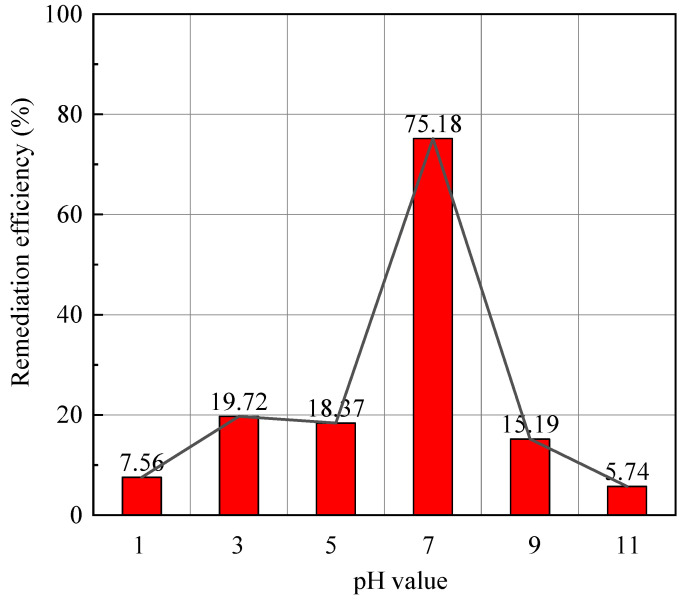
Healing efficiency of microcapsules in various water environments.

The healing efficiency of microcapsules in solution of various pH was studied. The pH values were 1, 3, 5, 9, and 11. The signals of frequency lower than 2.5 Hz and higher than 30 Hz were ignored to eliminate the interference during PZT tests. The frequency spectrum of microcapsules in water environments of various pH value is shown in Figure 14. The comparison and statistics of the healing efficiency of microcapsules in various water environments are shown in Figure 15 and Table 7.

The statistical results in Table 7 indicated that the healing efficiency of microcapsules on CCS varied obviously with the pH values. The healing efficiency of microcapsules in freshwater was up to 75.18%, which were 5.74% to 19.72% in the acid and alkali environment. The self-healing efficiency decreased with the degree of acid and alkali of the surrounding water. In addition, there was a large difference between the healing efficiency in water of pH 5 and pH 7, as well as between pH 9 and pH 7. In fact, the essential difference between pH 5 versus pH 7 and pH 9 versus pH 7 was the difference between electrolyte corrosion (in acid and alkali solutions) and absence (in freshwater) but not only the degree of the pH. It was suggested that the reaction of the core materials of the microcapsule, the epoxy resin and curing agent, were affected by the pH value of the water environment. Consequently, the healing effect of microcapsules was significantly inhibited by acid and alkali solutions. The peracid and peralkalic water environment would significantly inhibit the healing effect of the microcapsules. The healing efficiency of microcapsules in seawater was 3 to 10 times that in the acid–base environments. It was concluded that the microcapsule-based CCS were suitable for working in freshwater and in the seawater of low healing requirements.

## 5. Conclusions

The mechanical behavior and self-healing efficiency of microcapsule-based CCS were investigated through a series of UCS, SHPB, and PZT tests. The effect of microcapsules on the dynamic and impact mechanical properties of CCS were revealed by the uniaxial compression tests and SHPB tests. The energy of CCS before and after healing was analyzed through PZT tests. In addition, the healing efficiency of microcapsules in freshwater, seawater, and water of various pH was further discussed. The major conclusions can be summarized as follows:

1. The microcapsule in the CCS could improve the initial strength of the specimens by 45–83%. The optimal mixed ratio of microcapsule was 3% (i.e., mass ratio to the cement) under the synthesized rotating speed of 450 rmp. The seawater environment would decrease the compressive strength of the microcapsule-based CCS.
2. The SHPB impact tests indicated that the mix of microcapsules improved the initial dynamic compressive strength of the CCS. The absorbed energy by microcapsules was dissipated in the damage and deformation of the CCS specimens and the heat change of microcapsules. The microcapsule would reduce the dependence of the strength of the specimen on the incident energy and reduce the growth rate of strength with the energy density.
3. According to the PZT test results, the healing efficiency of microcapsules in freshwater and seawater was 75% and 59.56%, respectively. The acid–base environment of the surrounding water of microcapsule-based CCS would inhibit the healing effect of microcapsules. The healing efficiency in acid–base water ranged from 5.74% to 19.72, which was especially low in the peracid and peralkalic water environment.

## Figures and Tables

**Table 1:** Coral sand particle grading index.

0.25–0.5 (mm)	0.5–1 (mm)	*d*_10_ (mm)	*d*_50_ (mm)	*d*_90_ (mm)	*C_u_*	*C_c_*
43.62%	56.38%	0.29	0.40	0.61	2.10	0.90

**Table 2:** Component of artificial seawater.

NaCl (g/L)	MaCl_2_ (g/L)	Na_2_SO_4_ (g/L)	CaCl_2_ (g/L)	H_2_O (L)
24.53	5.20	4.09	1.16	1

**Table 3:** Synthesis materials of microcapsule.

Usage	Reagent	Purity Specifications
*Core material*	Epoxy Resin (E-51)	Technical pure
*Wall material*	Carbamide	Analytically pure
	Methanal	Analytically pure/ 37% aqueous solution
*pH Regulator*	Triethanolamine	Analytically pure
	Anhydrous sodium carbonate	Analytically pure
*Emulsifier*	Sodium dodecyl benzene sulfonate	Analytically pure
	Polyvinyl alcohol (PVA)	Analytically pure
*Defoamer*	N-caprylic alcohol	Analytically pure
*Acid catalyst*	Ammonium chloride	Analytically pure
*Curing agent*	Resorcin	Analytically pure

**Table 4:** Specimen preparation scheme.

Test	Specimen ID	Pre-Treatment	Microcapsule Content (%)	Rotating Speed (rpm)	Mixing Water
Uniaxial compressive test	U-1	No	0	-	Seawater
	U-2		0	-	Freshwater
	U-3		1	150–800	Freshwater
	U-4		3	150–800	Freshwater
	U-5		5	150–800	Freshwater
	U-6		7	150–800	Freshwater
	U-7		9	150–800	Freshwater
	U-8		3	450	Seawater
SHPB test	S-1	No	0	-	Freshwater
	S-2		3	450	Freshwater
	S-3		3	450	Seawater
PZT test	P-1	Yes	3	450	Seawater
	P-2 (pH = 1)				Freshwater
	P-3 (pH = 3)				Freshwater
	P-4 (pH = 5)				Freshwater
	P-5 (pH = 7)				Freshwater
	P-6 (pH = 9)				Freshwater
	P-7 (pH = 11)				Freshwater

**Table 5:** Parameters of SHPB test.

Parameter	Value
Launch pressure (P*_l_*)	0.6, 0.8, 1.0, 1.2 Bar
Wave velocity of impact bar (*C*_0_)	5000 m/s
Elastic modulus of impact bar (*E*_0_)	210,000 MPa
Diameter and length of bullet (*D_b_* and *L_b_*)	60 mm and 50 cm
Length of incident bar and transmission bar (*L_i_* and *L_t_*)	2.8 m
Diameter of incident bar and transmission bar (*D_i_* and *D_t_*)	60 mm
Frequency of dynamic acquisition instrument (*F*)	2.5 MHz
Amplification factor of dynamic acquisition instrument (*F_a_*)	100
Bridge voltage (*V_b_*)	2 V

**Table 6:** Failure modes under different strain rate.

Strain Rate	115 s^−1^	165 s^−1^	214 s^−1^	251 s^−1^
With microcapsule	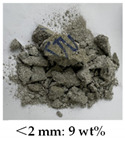	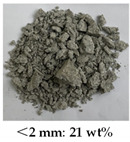	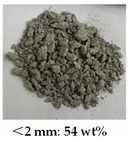	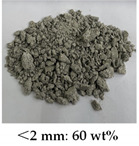
Without microcapsule	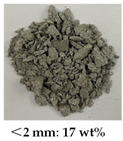	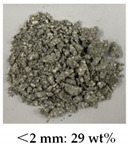	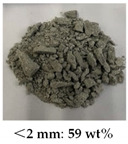	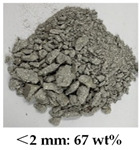

**Table 7:** Comparison of healing effect of microcapsules in various water environments.

Group	pH = 1	pH = 3	pH = 5	pH = 9	pH = 11
Initial wave velocity (m/s)	1235.03	1210.67	1220.45	1254.99	1275.49
Post-damage (m/s)	943.47	886.86	994.57	875.19	821.17
Post-healing (m/s)	1081.54	1120.30	1177.25	1156.77	1031.23
Initial energy (mJ)	7.7495	8.3060	6.9290	5.7970	4.0750
Post-damage energy (mJ)	0.4569	0.2360	0.5160	0.4360	0.6930
Post-healing energy (mJ)	1.0088	1.8280	1.6940	1.2920	0.8870
Healing efficiency (%)	7.56	19.72	18.37	15.97	5.74

## Data Availability

In this study there is no data used.
